# Creative health assets in Staffordshire: Harnessing community power to tackle health inequalities

**DOI:** 10.1177/17579139261462489

**Published:** 2026-08-01

**Authors:** R Marsden

**Affiliations:** Research Fellow in Creative-Public Health, NIHR SPHR Transdisciplinary Fellowship, Department of Applied Health Sciences, School of Health Sciences, College of Medicine and Health, University of Birmingham, Birmingham B15 2TT, UK

**Keywords:** public health, health inequalities, creative health, arts-based interventions, community assets, patient and public involvement and engagement

## Abstract

**Aims:**

This report investigates the role of creative health assets in addressing health inequalities across Stoke-on-Trent and Staffordshire. The project aimed to map and align arts-based interventions delivered by voluntary, community, faith and social enterprise (VCFSE) organisations with public health priorities and to explore how these assets respond to hyperlocal needs and contribute to community health and wellbeing.

**Methods:**

A scoping exercise was conducted from January to May 2024 as part of a Transdisciplinary Placement (TDP) within the National Institute for Health and Care Research (NIHR) School for Public Health Research (SPHR). The methodology included a literature and strategy review, analysis of public health data and two online engagement sessions with the NIHR SPHR’s Public Health RESearch for Health (PHRESH) Public Advisory Committee (PAC). Through desk-based research and network engagement, over 100 creative health assets were identified, with Stoke Creates being a key partner and stakeholder.

**Results:**

Findings revealed the prevalent but under-recognised and underutilised landscape of creative health assets, including arts organisations, charities and cultural networks. PAC members highlighted the benefits and barriers to engagement, including financial constraints, cultural competency gaps and limited visibility of opportunities. Strategic disconnects between cultural and public health policies were identified, alongside opportunities for future integration. Overall, these assets play a vital role in promoting mental and physical wellbeing, fostering social cohesion and addressing social determinants of health.

**Conclusion:**

Creative health assets and arts-based interventions offer significant potential to reduce health inequalities and improve public health outcomes in Stoke-on-Trent and Staffordshire. Greater recognition, investment and strategic alignment are needed to embed creative public health approaches within local governance and health systems. Future work will focus on comprehensive asset mapping including associated health outcomes and impact, research on the role of heritage and green-blue infrastructure, and the development of a creative health network for the region.

## Plain Language Summary

Creativity and community for better health in Staffordshire

This project looked at how creativity and the arts can help improve people’s health and wellbeing in Staffordshire and Stoke-on-Trent, areas where many people face serious health challenges. It focused on organisations and places, like community centres, theatres, museums and charities that use creative activities to support health. These are called creative health assets. The research was part of a placement funded by the National Institute for Health and Care Research (NIHR). It involved reviewing local health data, public health and cultural strategies, and identifying over 100 creative health organisations across the region. The project also included two group sessions with members of the public, where people shared their experiences of creative activities and how these helped their mental and physical health. People described creative health as empowering, calming and a way to connect with others. Activities like gardening, music, walking and crafts were seen as important for wellbeing. However, many also said that cost, lack of awareness of activities and cultural barriers made it hard to access these opportunities. The findings show creative health assets are a valuable but often overlooked part of public health. They can help reduce health inequalities and support people in ways that traditional healthcare might not. The next phase of the project will focus on mapping these assets more fully, exploring how nature and green spaces, and heritage, can be part of creative health, and working with local organisations to build a stronger network. Creativity is about arts, health and community towards better health for everyone.

## What is Creative-Public Health?

Creative health is broadly defined as an interdisciplinary field exploring the relationship between the arts, culture, creativity, health, care and wellbeing. Its primary aim is to improve health and social care and wellbeing outcomes, contributing to ‘the prevention of ill health, promotion of healthy behaviours, management of long-term conditions, and treatment and recovery across the life course’.^1^ Holistic, person-centred and equity-focused, it recognises ‘the arts are integral to health and healthcare provision and that everybody, regardless of their health status, should have access to creative opportunities’.^2^

This report defines ‘creative-public health’ as the integration of arts and culture into public health systems to enhance community engagement, tackle health inequalities and address the social determinants of health. It is about creating inclusive conditions and opportunities for arts, creativity and culture to be embedded in the health of the public so it is available and accessible to all,^1^ while making health literacy more accessible, inclusive and culturally relevant, particularly in underserved areas, in the case of this project, Staffordshire and Stoke-on-Trent. Spanning community choirs supporting people with Parkinson’s to theatre performances exploring chronic illness through lived experience, these public health interventions illustrate how creative-public health can provide non-medical routes to health and social care, furthermore, empowering communities to take ownership of their health through self-management.

## Creative Health Assets as Non-Traditional Providers (NTPs)

Creative health assets, also referred to as community assets, ‘Assets of Community Value (ACV)’ or community health assets, are people, places, services, or organisations that use arts and culture to support health and wellbeing.^3^ They serve as hubs providing essential services to tackle health inequalities and can be individuals, physical structures, land, community services or businesses, such as community centres, galleries, theatres, libraries, churches, allotments and a wide range of voluntary, community, faith and social enterprise (VCFSE) organisations. These assets play a vital role in improving quality of life, enhancing social wellbeing and social interests (e.g. culture, hobbies, sports, exercise) and addressing the social determinants of health through creative and cultural engagement.^3^ By delivering arts-based interventions such as theatre, music, visual arts and nature-based activities, they not only support the physical and mental health of individuals and communities but also foster social interaction, belonging and community cohesion.

Asset-based community development values the abilities, capacity, skills, knowledge, connections and potential of communities, empowering them rather than focusing on deficits.^4^ The National Health Service (NHS) has recognised this role of ‘non-traditional providers’ (NTPs) in supporting people with long-term conditions, improving outcomes, reducing pressure on health and social care services, and enabling more cost-effective resource use.^5^ However, asset-based approaches may not always align with the priorities of health services or local authorities, as communities often identify different issues as most pressing.^4^ Within this context, creative health assets remain under-recognised in mainstream public health strategies – as will be discussed later in this report – despite their capacity to harness community strengths and respond more effectively to local and hyperlocal needs than statutory services.

## Health Inequalities in Stoke-on-Trent and Staffordshire

Stoke-on-Trent is among the 20% most deprived districts in England, with over half its population living in disadvantaged circumstances.^3^ Life expectancy in the city is up to 13 years lower in its most deprived areas compared to its more affluent ones.^6^ The city also records higher-than-average rates of unemployment, infant mortality, obesity, smoking and poor mental and physical health. By contrast, Staffordshire is healthier overall; however, it faces challenges of an ageing population, rising rates of chronic conditions such as diabetes, chronic obstructive pulmonary disease and dementia, and pockets of deprivation.^7^ These disparities highlight the need for targeted, community-driven and place-based interventions to address persistent health inequalities and bridge the health gap between Stoke-on-Trent and Staffordshire.

Here, creative health assets can play a central role in this response, ‘contributing to the building blocks for good health’,^3^ where a new approach to public health is emerging – one that harnesses the power of creativity, community and placemaking. This shift reflects a growing recognition that arts-based interventions and cultural engagement can play a vital role in prevention, improving wellbeing and addressing the social determinants of health.^8^

*Mapping Creative Health Assets: Mobilising Arts-based Interventions to Tackle Health Inequalities in Staffordshire* (2024) explored how creative health assets can be mobilised to address disparities, tackle and reduce health inequalities and improve health and wellbeing outcomes across the region. The project was conducted as part of a ‘Transdisciplinary Placement (TDP)’, an initiative funded and supported by the NIHR School for Public Health Research (SPHR) and hosted by the PHRESH (Public Health RESearch for Health) consortium, a partnership between the University of Birmingham, University of Warwick and Keele University. The researcher was based at the University of Birmingham for the duration of the placement. TDPs are short, intensive research contracts – typically 6 to 8 weeks – that provide professionals from outside traditional public health backgrounds the opportunity to bridge academic, applied and cross-sector approaches. They are designed to support exploratory work on pressing health challenges and generate innovative, system-level insights that might not emerge through conventional disciplinary routes.

This TDP was aligned with two SPHR research programmes ‘Health Inequalities’ and ‘Healthy places, Healthy Planet’, situating the project within broader national public health priorities. The project involved a scoping exercise comprising a brief literature and contextual review into the emerging field of ‘creative-public health’, analysis of public health data for Stoke-on-Trent and Staffordshire, and a comparative review of local cultural and public health strategies. A desk-based mapping exercise identified over 100 creative health assets across the region, including arts organisations, charities, museums, libraries, and community interest companies (CICs), many of which deliver arts-based interventions with potential health impacts. Crucially, two online engagement sessions with the PHRESH Public Advisory Committee (PAC) were facilitated to gather lived experience perspectives on creative health. These sessions revealed how individuals define, engage with and benefit from creative activities, while also highlighting barriers to access and participation and perspectives of healthcare systems.

Together, the findings present a case for embedding creative health into regional policies, strategies and everyday lives, positioning creativity not as a peripheral activity, rather central to inclusive and place-based and creative-public health practices, and empowering communities.

## PHRESH PAC Insights

An important facet of the TDP project was two online engagement sessions delivered through the PHRESH PAC, an established group of approximately 30 public contributors from across the West Midlands and wider UK. Membership is voluntary, and individuals join based on their interest in public health and willingness to contribute to lived experience research. Members were not required to have prior understanding of, or participation in, informal or formal creative health assets or services. Therefore, the group includes individuals who engage informally in creative activities (not identified as creative health) and formal creative health services and arts-based interventions.

PAC predominantly meets online and brings together individuals with varied lived experience of health, illness, treatment, management and recovery, across age profiles from younger to older adults: 32% aged 20–30, 18% aged 40–50, 36% aged 50–60, 5% aged 60–70 and 9% aged 70+; however, no members fall in the 30–40 age range. The group are ethnically diverse, comprising 56% White British, 4% White other, 8% Asian/Asian British, 4% Black/Black British, 16% mixed, 4% other and 8% prefer not to say. Nearly half of members – 48% – have caring responsibilities, 58% identified as heterosexual and 44% identified as having a disability. Together, these characteristics ensured varied levels of engagement and a breadth of perspectives, including those who might be at increased risk of health disparities and with differing access to creative health assets.

Two online engagement sessions were organised and accessed internally through the University of Birmingham, co-facilitated by the researcher and the PHRESH Programme Manager, Lucy Oakey. They were delivered via Microsoft Teams, with each session lasting 30 minutes:

- Session 1 (8 March 2024) involved 20 participants (75% female, 25% male). Prompts explored everyday creative activities, community-based provision, perceived benefits and experiences of creative health (questions 1–5 below);
- Session 2 (29 April 2024) involved 12 returning participants (83% female, 17% male). This session built directly on the first session and focused on definitions of creative health, perceived impacts on health and social care, and barriers to access (questions 5–8).

Participants received the discussion questions in advance of the sessions:

1. What creative activities do you like doing / enjoy?
2. Are there creative activities happening in your local community?
3. What is good about these creative activities?
4. How do creative activities help health services?
5. What are your experiences of creative health?
6. What are the benefits of doing creative activities for your physical and / or mental health?
7. How do you define/understand ‘creative health’?
8. How can creative activities help and support social care, healthcare and wellbeing services?

Many participants described engaging in a wide range of creative activities including gardening, cooking, walking, textile arts, music (live, playing, singing) and photography; however, most did not recognise these as ‘creative health’. Their participation was largely informal, self-directed and intrinsically woven into daily routines as a source of calm, connection and self-expression. Here, creative health is practised without being recognised as such, rather than accessed through creative health services or arts-based interventions delivered via community or creative health assets.

Across both sessions, participants characterised creative activities as ‘a coping mechanism’, ‘a stress-removing exercise’, ‘a way to recharge’ and ‘empowering and energising’. Others reflected in their therapeutic value stating, ‘Creative activities should be a key component of therapy’, while another noted, ‘It brings some joy [. . .] it’s really important for mental health’. As participants described, it ‘stops me thinking about everything else in my life’, ‘give you a certain sort of calmness’ and serve as a moment to ‘empty your head’. These reflections highlight creative activities as deeply personal and socially embedded, supporting individual resilience and collective wellbeing.

All these [creative] activities – whether done alone or in a group – are a fabulous way to improve mental health and wellbeing. (PAC member)

At this stage, the creative health benefits of participating in creative activities were clearly identified by the group. Although personal engagement with creative activities was highly valued, participants recognised these do not substitute the structured, facilitated and socially connected, group benefits offered by creative health assets, particularly for those who may lack confidence, resources or social networks to engage independently.

Yet, participants also noted that while creative activities have the potential to be ‘part of health and wellbeing services, in practice that’s not really how it is’. This reflects a broader perception that the creative health benefits of creative activities and arts-based interventions are not fully recognised and remain undervalued within health systems. Participants further emphasise a persistent gap, specifically between the aspiration of ‘thinking holistically’ and the realities of health and social care delivery, suggesting the need for more interdisciplinary and collaborative models of public health practice, in the case of this project, towards models of creative-public health.

Barriers to access and participation were also evident. Cost was the most frequently cited challenge, with many creative activities, including participation in creative health services and arts-based interventions, become unaffordable due to rising cost of living expenses. Accessibility, cultural competency and digital exclusion were also significant concerns, alongside the short-term nature of creative health programmes. One participant reflected, ‘Imagine sitting in a corner not being able to take part due to not speaking the local language. It means isolation [. . .] the opposite of recovery’. Another added, ‘Equitable access just sadly doesn’t exist’. These insights emphasise the urgent need for inclusive, affordable and culturally relevant creative health programmes, backed by appropriate training, sustainable investment, resources and infrastructure.

Participants also emphasised the importance of community connectors and link workers in making creative health assets visible and accessible across diverse communities. Although not all participants were familiar with formal link worker roles, many had encountered similar figures informally, such as community organisers, charity volunteers or group facilitators. This signals to the potential for formalised pathways, for example, through social prescribing, to increase access to creative health, particularly for those not currently engaged. At the same time, engagement with creative health assets also inspired some participants to take proactive steps within their communities. Several had established support groups, often in collaboration with charities, or partnered with local organisations to deliver activities. Others had taken on leadership roles by chairing local groups, joining public and patient involvement and engagement (PPIE) groups, or becoming local governors within community healthcare trusts. These examples demonstrate the precariousness of the creative health sector – often reliant on voluntary labour – yet its transformative potential to empower individuals as community leaders and changemakers.

## Mapping Creative Health Assets

A desk-based mapping exercise using secondary research followed a structured, iterative search process, drawing on publicly accessible sources from arts, culture, heritage and VCFSE organisations; regional and national creative health directories; NHS Trust, Integrated Care Board (ICB) and local authority websites; social media platforms, and grey literature. Search terms focused on geographical location (‘Staffordshire’, ‘Stoke-on-Trent’, ‘Staffordshire Moorlands’) and creative health terminologies (‘arts and health’, ‘arts in health’, ‘arts for health’, ‘creative health’, ‘creative wellbeing’, ‘creative health asset’, ‘community asset’). Creative health assets – whether informal or formal – were included if they delivered or facilitated a creative activity with potential health or wellbeing outcomes and impacts, were located in Staffordshire, had a public-facing presence and demonstrated active or recent provision in the previous 6 months.

**Figure 1:**
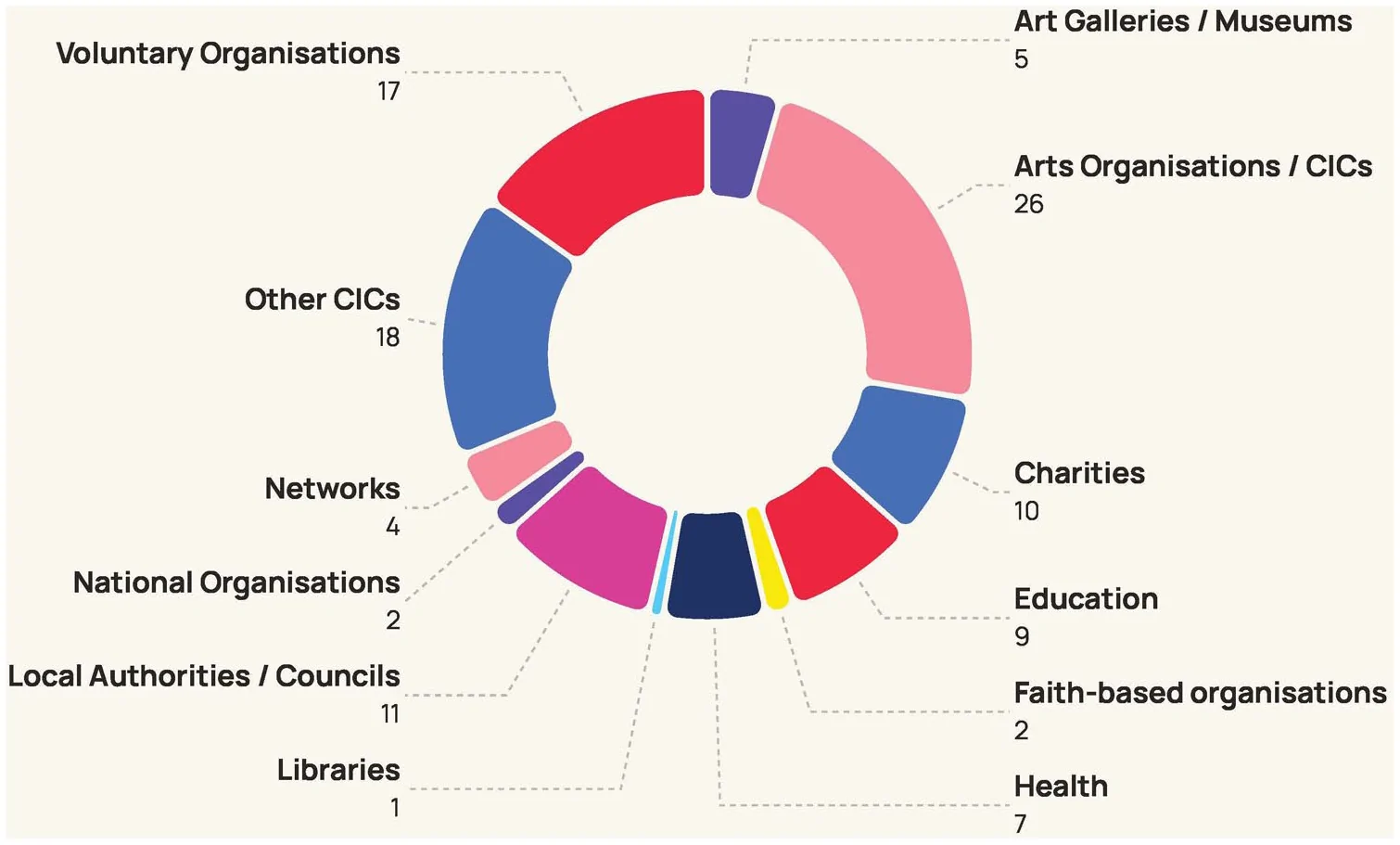
Creative health assets in Stoke-on-Trent and Staffordshire, May 2024.

A total of 112 creative health assets were identified as delivering arts-based interventions across Stoke-on-Trent and Staffordshire. These assets span a wide range of sectors, including art galleries and museums, arts organisations, CICs, charities, educational and faith-based organisations, health services, libraries, local authorities, local networks and national organisation affiliation (Figure 1). Arts organisations and CICs, alongside voluntary organisations, emerged as the most prevalent asset types. The *Connected Communities Impact* report echoed overall findings, with participants attending the *Staffs CAN (Community and Civic Advisory Network)* noting they were ‘surprised at the volume of projects that support health and wellbeing’,^9^ calling for greater visibility, inclusivity and outreach to harder-to-reach groups.

This snapshot represents only a fraction of the 638 artists and arts organisations recognised by the Stoke-on-Trent and North Staffordshire Cultural Education Partnership.^10^ Even though assets operate across multiple categories, they have only been counted once. This reflects how creative health assets can have a greater awareness of a community’s strengths and are more responsive to hyperlocal needs through cross-sector working.^4^ *Stoke Creates*, the city’s ‘Cultural Compact’, emerged as a key stakeholder in this space, acting as an inclusive catalyst, facilitating partnerships and collaboration between artists, cultural organisations and public health stakeholders. Committed to place-based regeneration, *Stoke Creates* works to amplify the benefits of creative and cultural engagement in the region.^11^

One strand of its programme, the ‘Stoke Cultural Exchange Forum (SCEF)’, provides a launchpad bringing together the city’s freelance artists and cultural stakeholders to connect, network, share resources, access training and collaborate on commissions to ensure a vibrant, diverse and highly skilled arts and cultural sector.^11^ Partnering with *Stoke Creates* led to the inaugural ‘Creative Health Exchange Forum’ in September 2024, bringing together practitioners, researchers and community members to explore the future of creative-public health in the region.

## Strategic Gaps and Opportunities

Despite the benefits evidenced by PAC members and the prevalence of creative health assets identified in the region, the TDP project also highlights a disconnect between cultural and public health strategies in Stoke-on-Trent and Staffordshire. Public health frameworks including the *Stoke-on-Trent Joint Health and Wellbeing Strategy* (2021–2025) and the *Staffordshire Health and Wellbeing Strategy* (2022–2027) reference community assets but rarely integrate the cultural sector or recognise arts-based interventions.^6,12^ Conversely, *Stoke-on-Trent’s Cultural Strategy* (2022–2028) acknowledges creativity’s role in supporting wellbeing but pays little attention to health inequalities.^10^ In contrast, Birmingham City Council has integrated creative-public health into its ‘Bolder Healthier City’ strategy,^13^ exemplifying a model Stoke-on-Trent and Staffordshire could adapt to strengthen their public health practice.

With a new ‘Cultural Strategy for Staffordshire’ currently in development, there is a timely opportunity to embed creative health more meaningfully. Bridging this gap requires embedding creative health within governance structures, through mechanisms such as a dedicated Creative Health Board, integration into Health and Wellbeing Boards or a regional creative-public health strategy. To achieve this, longer-term and stronger collaboration between the VCFSE sector, public health teams and cultural organisations will be essential. Events such as the ‘Creative Health Exchange Forum’ could help facilitate this work – an opportunity that can only be realised through sustained financial and resource investment.

## The Power of Placemaking

As the TDP project transitions into its next phase, it aims to deepen understanding of creative health through comprehensive asset mapping. Combining geographical, community and artistic approaches, this work will visualise and connect creative health infrastructure, enabling stakeholders to leverage assets more effectively and foster more integrated approaches to health, care and wellbeing. A particular focus will be on placemaking and Green Blue Infrastructure (GBI).^14^ Stoke-on-Trent, home to 96 parks and green spaces,^3^ and one of the highest health index scores for access to green space,^15^ offers unrealised potential for creative and nature-based interventions.

Both the *Stoke-on-Trent Cultural Strategy 2022–28*^10^ and local Health and Wellbeing Strategies highlight the cultural and social value of public places, parks, green and rural spaces.^6,12^ Interestingly, PAC members consistently celebrated outdoor activities and connecting with nature and wildlife as among their most enjoyable creative activities. Yet, as Kirby and Scott state, ‘the evidence of health and wellbeing impacts of GBI is not yet fully understood, hindering its wider adoption in health and environmental practice’.^14^ Concurrently, the role of heritage must not be overlooked. Stoke-on-Trent and Staffordshire’s rich cultural history, particularly its ceramics and craft traditions, are central to the region’s identity and creative infrastructure. The recent designation of Stoke-on-Trent as a *World Craft City* by the World Crafts Council in 2024 highlights the global significance of its heritage. This recognition presents a unique opportunity to integrate heritage-based creative health interventions into public health strategies, further strengthening place-based approaches to prevention, health and wellbeing.

In a region where health inequalities persist, the TDP project makes an urgent case for embedding creative-public health in Stoke-on-Trent and Staffordshire. It demonstrates creative health assets are not peripheral, rather central to building healthier, more resilient communities. By listening to PAC members, mapping assets and fostering interdisciplinary collaborations, the project establishes a framework for what comes next – further research into the health outcomes and impact of the creative health assets identified, and action towards more inclusive, responsive and holistic public health strategies and systems. We should all have the opportunity to access and benefit from creative health. As one PAC member shared, ‘I think everyone should have something creative in their lives [. . .] it’s very fulfilling and there’s something for everyone’.
